# Mutation profile of acute myeloid leukaemia in a Chinese cohort by targeted next‐generation sequencing

**DOI:** 10.1002/cnr2.1573

**Published:** 2021-10-06

**Authors:** Benny Man Wai Lit, Belinda B. Guo, Jacques A. J. Malherbe, Yok Lam Kwong, Wendy N. Erber

**Affiliations:** ^1^ Department of Pathology Queen Elizabeth Hospital Hong Kong China; ^2^ School of Biomedical Sciences The University of Western Australia Perth WA Australia; ^3^ Medical School The University of Western Australia Crawley WA Australia; ^4^ Department of Medicine Queen Mary Hospital, The University of Hong Kong Hong Kong China; ^5^ PathWest Laboratory Medicine Nedlands WA Australia

**Keywords:** Chinese AML, *DNMT3A*, mutation frequency, next‐generation sequencing

## Abstract

**Background:**

Acute myeloid leukaemia (AML) results from the clonal expansion of blast cells of myeloid origin driven by genomic defects. The advances in next‐generation sequencing (NGS) have allowed the identification of many mutated genes important in the pathogenesis of AML.

**Aims:**

In this study, we aimed to assess the mutation types and frequency in a Chinese cohort presenting with de novo AML cohort using a targeted NGS strategy.

**Methods:**

In total, we studied samples from 87 adult patients with de novo AML who had no prior history of cytotoxic chemotherapy. Samples were evaluated using a 120‐gene targeted NGS panel to assess the mutation profile.

**Results:**

Of the 87 AML patients, there were 60 (69%) with a normal karyotype. 89.7% of patients had variants, with an average of 1.9 mutations per patient (range: 0–5 mutations per patient). *DNMT3A* variants were the most common, being detected in 33 patients (37.9%). *NPM1* (34.5%), *IDH1/2* (24.1%) and *FLT3‐ITD* (20.7%) mutations was the next most common. Of the patients with *DNMT3A* mutations, 24.2% also had mutations *NPM1* and *FLT3‐ITD* and 6.1% *NPM1*, *FLT3‐ITD* and *IDH* mutations.

**Conclusion:**

Both *DNMT3A* and *NPM1* mutations were more common than in other Chinese and Western AML cohorts that have been studied. *DNMT3A* mutations tended to co‐occur with *NPM1* and *FLT3‐ITD* mutations and were most commonly seen with a normal karyotype.

## 1 INTRODUCTION

Acute myeloid leukaemia (AML) is a group of heterogeneous diseases characterised by distinct clinical, morphological, cytogenetic and genetic features. AML is classified as a unique disease entity in the current 2017 World Health Organization (WHO) classification of tumours of haematopoietic and lymphoid tissues^1^, ^2^, ^3^ based on specific recurring genetic abnormalities for predicting prognosis and treatment response. Specific mutations that are included in the classification are *NPM1* and *CEBPA*. Recurrent chromosomal structural abnormalities are now confirmed as diagnostic and prognostic markers, which indicate that acquired genetic abnormalities are essential in leukaemogenesis.^4^, ^5^, ^6^ However, many cytogenetically normal AML (NK‐AML) cases that lack recurrent structural abnormalities, and account for 40% to 50% of de novo adult AML,^7^ are associated with an intermediate clinical outcome.^2^ It is known that the prognosis of AML patients is significantly affected by multiple genetic mutations and many leukaemias are composed of multiple subclones with different responses to treatment protocol.^8^ In the Cancer Genome Atlas (TCGA) group, 23 significantly mutated genes were identified by whole‐genome or whole‐exome sequencing in 200 AML patients. These include genes that are well known to be related in AML pathogenesis (e.g., *DNMT3A*, *FLT3*, *NPM1*, *IDH1*, *IDH2* and *CEBPA*).^6^ For AML risk classification, it is important to study the mutations in multiple genes simultaneously because of complicated interactions with different pathways in leukaemogenesis.^9^, ^10^

The majority of data regarding the frequencies of specific AML mutations have been derived from Caucasian populations.^6^, ^11^, ^12^ These have shown frequencies for the most common variants ranging from 26.3% to 33.0% for *FLT3*, 23.0% to 28.0% for *NPM1* and 22.1% to 26.0% for *DNMT3A*. Other populations groups that have been studied are Japanese, Korean and Singaporean.^13^, ^14^, ^15^ The genes that are involved in AML in these populations are the same, but with some differences in frequency. Few studies have assessed AML mutations in Chinese populations.^16^, ^17^, ^18^ These reports have shown the mutational frequencies ranging from 12.5% to 14.0% for *DNMT3A* and 15.0 to 15.9 for *NPM1*. In the present study, we have progressed this by studying an adult Chinese population presenting with de novo AML using a next‐generation sequencing (NGS) platform. This targeted approach assessed the mutation profile and frequency of 120 genes associated with myeloid malignancies. The purpose of this study was to assess the frequency of mutations in the genes most commonly associated with aberrations in myeloid neoplasms in a population of Chinese patients with de novo AML.

## 2 MATERIALS AND METHODS

### 2.1 Patients and samples

In total 87 adult Chinese patients with de novo AML without any prior history of cytotoxic chemotherapy were studied. These patients were diagnosed with AML between 2004 and 2014 in two acute hospitals in Hong Kong and classified according to the 2001 and 2008 WHO classification.^2^, ^19^ Diagnoses were made on bone marrow morphology and immunophenotyping, using standard methods.

Cytogenetic analysis was performed on short‐term unstimulated synchronised culture using fluorodeoxyuridine (FdU) of the BM or PB samples. Karyotypes were analyzed after Giema‐banding and reported in the International System for Human Cytogenetic Nomenclature.^20^, ^21^ Patients were divided into favourable, intermediate and adverse risk groups according to their cytogenetic results and also the incorporation of molecular analyses as recommended by European LeukaemiaNet (ELN) in 2010.^21^, ^22^, ^23^

DNA extraction was performed on the buffy coat of PB or BM samples using AllPrep DNA/RNA Mini Kit (Qiagen, Valencia, California) according to the manufacturer's protocol. *FLT3‐ITD* mutations were analysed by conventional PCR based molecular method. *FLT3‐ITD* mutations in exons 14 and exon 15 were detected by PCR and *ITD* was then confirmed by sequencing as previously described.^24^ *NPM1* exon‐12 mutations were detected by PCR and then fragment analysis was performed by ABI 3130 genetic analyzer (Applied Biosystems). The results were analyzed with GeneMapper Software Version 4.0 (Applied Biosystems) as previously described.^25^

### 2.2 Massively parallel sequencing

Ion AmpliSeq Designer was used to create an Ion AmpliSeq Custom Panel (Thermo Fisher Scientific). Twenty nanograms of DNA was used to create the amplicon library for sequencing the whole exons of 120 genes (Appendix S1) that are involved with myeloid disorders as previously described.^26^ The completed library was prepared using an Ion AmpliSeq Library Kit 2.0 and the custom primer panel (Thermo Fisher Scientific) following the manufacturer's instruction. Barcoded libraries were measured using the Qubit dsDNA HS Assay Kit and the Qubit 2.0 Fluorometer (Thermo Fisher Scientific). The barcoded libraries were clonally amplified by emulsion PCR (E‐PCR) on Ion Sphere Particles (ISPs) using an Ion PI Template OT2 200 Kit v2 and the Ion OneTouch 2 System (Thermo Fisher Scientific). Enrichment of the template‐positive ISPs was achieved by the Ion OneTouch 2 ES (Thermo Fisher Scientific). Sequencing of the enriched ISPs was prepared on an Ion Proton Sequencer (Thermo Fisher Scientific) using Ion Proton I (PI) chip and Ion PI Sequencing 200 Kit (Thermo Fisher Scientific) as previously described.^26^ Torrent Suite Software Version 4.0 (Thermo Fisher Scientific) was used for signal processing, base calling, sequence alignment, variant calling and to generate run metrics. Variants were confirmed with Variant Caller Version 4.0 (Thermo Fisher Scientific) and align reads to the reference human genome build 19. Ion Reporter Version 4.0 (Thermo Fisher Scientific) was used to annotate the variants and detect amino acid changes and diagnostic significance.

The following filtering criteria were used for the NGS variant mutations: Intronic and exonic synonymous variants were removed while exonic non‐synonymous variants were retained for analysis. For polymorphic variants, a minor allelic frequency ≥ 1% and/or found in single nucleotide polymorphism (SNP) Database were excluded. In addition, a variant allele frequency (VAF) > 5% was used as the cutoff.

### 2.3 Statistical analysis

Chi‐square test was used to analyze the association between different gene mutations and also for other categorical variables. Two‐sided *p* value <.05 was considered statistically significant. All statistical analyses were performed with SPSS Version 26.0 (SPSS Inc, Chicago, Illinois).

## 3 RESULTS

### 3.1 Clinical and cytogenetic characteristics

There were 45 males and 42 females (M:F ratio = 1.07:1) newly presenting adult Chinese patients with de novo AML were included in the study. The mean age was 62 years (range: 20–91 years) with 39 patients (45%) being under the age of 60 years. Cytogenetic results showed 60 patients (60/87, 69.0%) with a normal karyotype (NK‐AML) and 27 patients (27/87, 31.0%) were abnormal (AK‐AML).

### 3.2 Mutation profile

**FIGURE 1:**
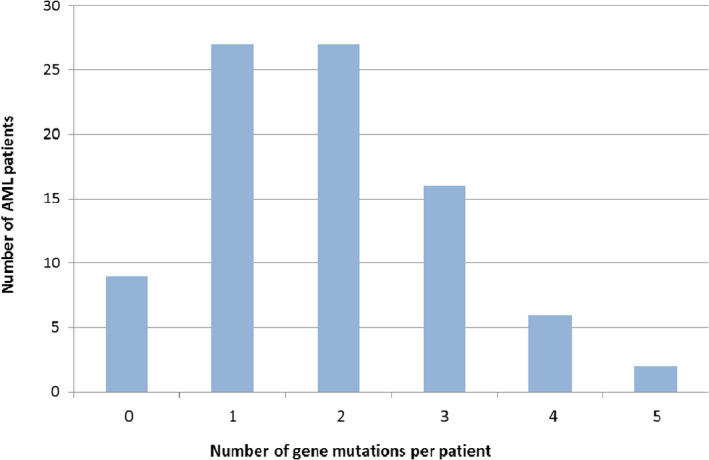
Number of gene mutations per patient of the acute myeloid leukaemia (AML) patient cohort

**FIGURE 2:**
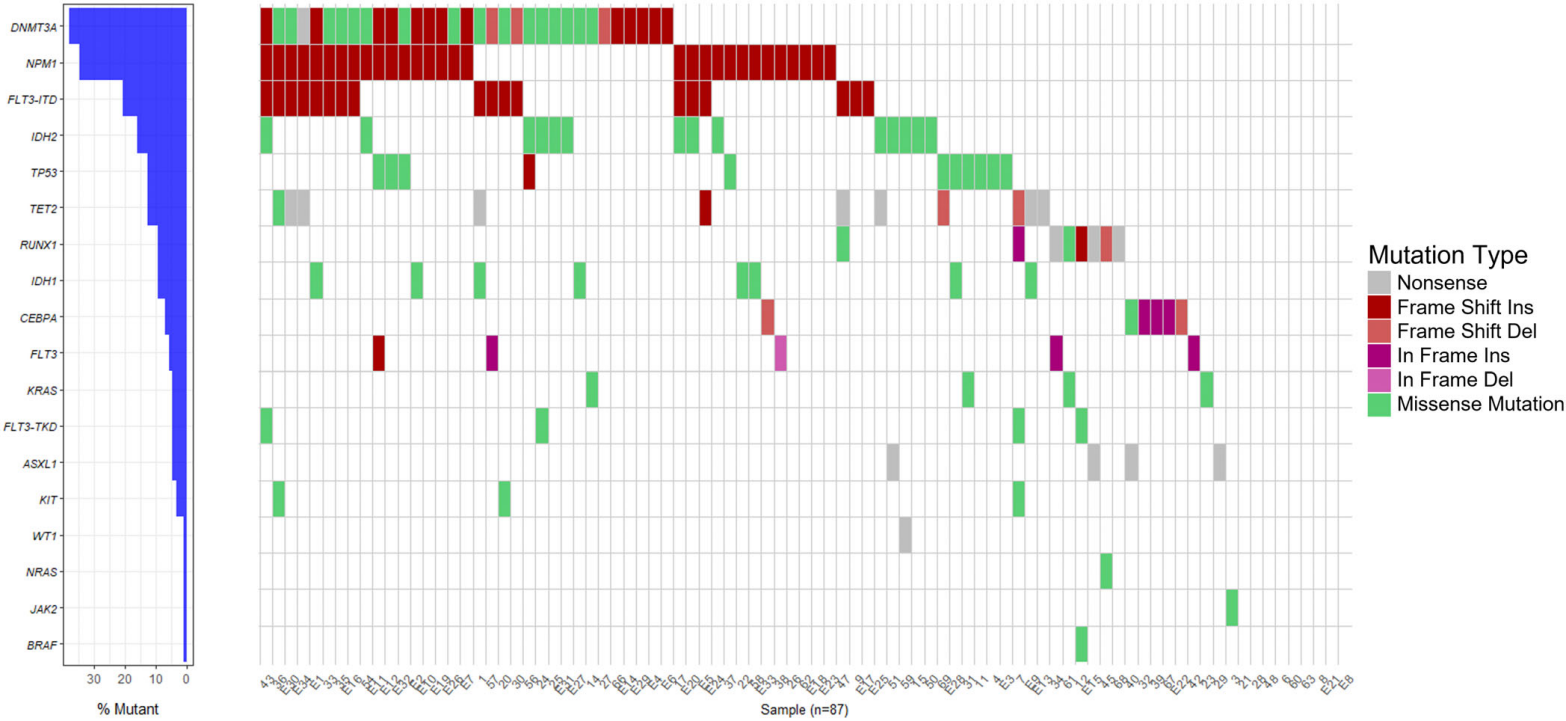
Waterfall plot of the filtered mutations in all patients. The left plot shows the frequency of each mutation. For clarity, *FLT3*‐*ITD* and *FLT3‐TKD* are shown separate to other mutations in *FLT3*. The right plot shows the types of mutations in each patient sample

**FIGURE 3:**
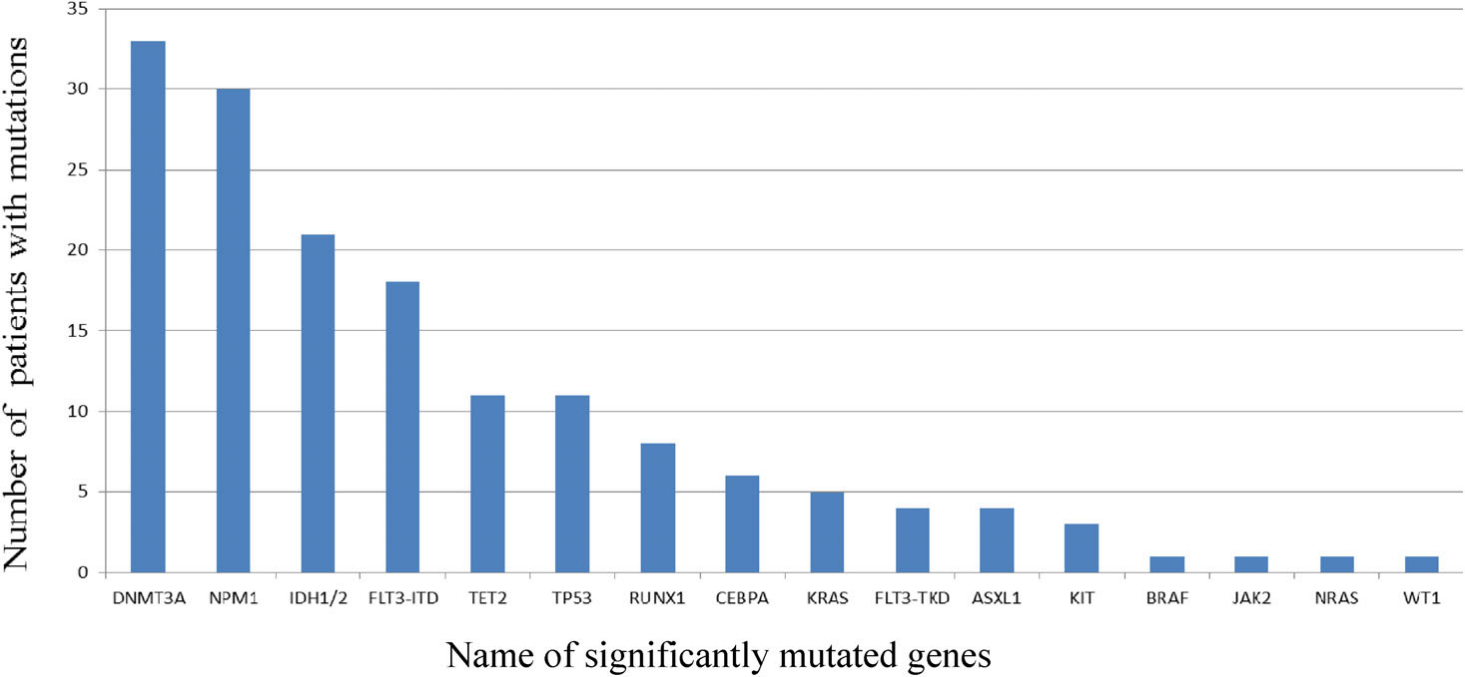
Mutation frequencies of significantly mutated genes in AML patients

After applying the filtering criteria, an average of 1.9 mutations were detected per patient (range: 0–5 mutations) and variants were seen in 78/87 (89.7%) of all patients (Figure 1). Of these 60/87 (69%) had a normal karyotype. Because of the lack of matched constitutional normal control material, we could not confirm that all gene mutations were somatic mutations because some may be rare SNPs. *DNMT3A* mutations were detected in 33/87 (37.9%) of patients (mean age 61 years). The other commonly mutated genes were *NPM1* (30/87, 34.5%), *IDH1/2* (21/87, 24.1%) and *FLT3‐ITD* (18/87, 20.7%) mutations (Figures 2, 3 and Table S1). Of the 33 patients with *DNMT3A* mutations (Table 1), 29 patients (29/33, 87.9%) had a normal karyotype. We also found that *DNMT3A* mutations had the highest frequency in NK‐AML patients (33/60, 55%). The most frequent mutations were located in R882, with R882H mutation being the most common (9/13, 69.2%), followed by R882C mutation (3/13, 23.1%). *DNMT3A* mutations tended to co‐exist with other mutations (Table 2). Among the patients with DNMT3A mutations, six patients had concomitant NPM1 and FLT3‐ITD mutations. In addition, two patients had IDH1/2 mutation that co‐occur with NPM1 and FLT3‐ITD mutations.

**TABLE 1:** Clinical and molecular features of acute myeloid leukaemia (AML) patients with *DNMT3A* mutations

Patient gender/age (years)	WHO/FAB diagnosis	Karyotype	*DNMT3A* mutations	*NPM1* mutations	*FLT3‐ITD* mutations	*IDH1/2* mutations
F/54	AML without maturation/AML M1	46, XX[18]	p.Arg882Cy (R882C)	Negative	Positive(57 base pairs duplication)	*IDH1*: p.Arg132His (R132H)
M/48	Acute myelomonocytic leukaemia/AML M4	46, XY[22]	p.Pro904leu (P904L)	Negative	Negative	Negative
M/78	AML with maturation/AML M2	46, XY[19]	p.Arg882His (R882H)	Negative	Positive (8 base pairs duplication)	Negative
F/54	AML with maturation/AML M2	47, XX,+?15[2]/46, XX[18]	p.Arg882His (R882H)	Negative	Negative	*IDH2*: p.Arg140Gln (R140Q)
F/46	AML without maturation/AML M1	46, XX[20]	p.Ser714cys (S714C)	Negative	Negative	*IDH2*: p.Arg172Lys (R172K)
F/70	Acute myelomonocytic leukaemia/AML M4	46, XX[19]	p.Lys299Asnfs (K299N)	Negative	Negative	Negative
F/53	AML without maturation/AML M1	47, XX, t(8; 21) (q22; q22),+15[19]	p.Lys299Asnfs (K299N)	Negative	Positive (39 base pairs duplication)	Negative
F/72	Acute monocytic leukaemia/AML M5b	46, XX[20]	p.Arg882 Pro (R882P)	p.Trp288Cysfs*12 (W288C) (mutation A with TCTG insertion)	Positive (18 base pairs duplication)	Negative
F/43	Acute myelomonocytic leukaemia/AML M4	46, XX[20]	p.Arg882 His (R882H)	p.Trp288Cysfs*12 (W288C) (mutation A with TCTG insertion)	Positive (48 base pairs duplication)	Negative
M/52	AML without maturation/AML M1	46, XY, t(2, 12) (p.23;q24.1)[20]	p.Cys710Tyr (C710Y)	p.Trp288Cysfs*12 (W288C) (mutation A with TCTG insertion)	Positive (42 base pairs duplication)	Negative
M/66	AML without maturation/AML M1	46, XY[18]	p.Glu545*	p.Trp288Cysfs*12 (W288C) (mutation A with TCTG insertion)	Positive (87 base pairs duplication)	*IDH2*: p.Arg140Gln (R140Q)
M/45	Acute myelomonocytic leukaemia/AML M4	46, XY[20]	p.Arg882Cys (R882C)	p.Trp288Cysfs*12 (W288C) (mutation A with TCTG insertion)	Negative	*IDH2*: p.Arg140Gln (R140Q)
F/62	AML without maturation/AML M1	46, XX[21]	p.Arg882His (R882H)	Negative	Negative	*IDH2*: p.Arg172Lys (R172K)
M/59	AML with maturation/AML M2	46, XY,t(10;11) (q23; p15)[1]/46, idem, add(6) (p25)[19]	p.Arg882His (R882H), p.Lys299Asnfs (K299N)	Negative	Positive (27 base pairs duplication)	Negative
F/70	AML with maturation/AML M2	46, XX[20]	p.Ser714Cys (S714C), p.Glu545*	Negative	Negative	Negative
F/78	AML without maturation/AML M1	46, XX[20]	p.Arg882His (R882H), P.Glu545*	p.Trp288Cysfs*12 (W288C) (mutation A with TCTG insertion)	Positive	*IDH1*: p.Arg132His (R132H)
M/65	Acute myelomonocytic leukaemia/AML M4	46, XY[20]	P.Glu545*	p.Trp288Cysfs*12 (W288C) (mutation A with TCTG insertion)	Negative	*IDH1*: p.Arg132His (R132H)
M/72	AML with maturation/AML M2	46, XY[20]	p.Trp893Val, p.Glu545*	Negative	Negative	Negative
F/87	AML with myelodysplasia‐related changes (multilineage dysplasia)	46, XX[20]	p.Glu545*	Negative	Negative	Negative
M/50	AML without maturation/AML M1	46, XY[20]	p.Arg882His (R882H), p.Glu545*, p.Trp893Valfs	p.Trp288Cysfs*12 (W288C) (mutation A with TCTG insertion)	Negative	Negative
M/80	AML without maturation/AML M1	46, XY[20]	p.Cys911Tyr (C911Y), p.Glu545*	p.Trp288Cysfs*12 (W288C) (mutation A with TCTG insertion)	Negative	Negative
F/49	AML without maturation/AML M1	46, XX[20]	p.Glu545*	p.Trp288Cysfs*12 (W288C) (mutation A with TCTG insertion)	Not done	Negative
F/82	AML with myelodysplasia‐related changes (multilineage dysplasia)	46, XX[20]	p.Glu545*	p.Trp288Cysfs*12 (W288C) (mutation A with TCTG insertion)	Negative	Negative
M/81	AML with maturation/AML M2	46, XY[20]	p.Glu545*	Negative	Negative	Negative
M/88	Acute myelomonocytic leukaemia/AML M4	46, XY[20]	p.Ser638Cys (S638C)	p.Trp288Cysfs*12 (W288C) (mutation A with TCTG insertion)	Positive	Negative
M/50	AML without maturation/AML M1	46, XY[20]	p.Thr503Asnfs (T503N)	p.Trp288Cysfs*12 (W288C) (mutation A with TCTG insertion)	Negative	Negative
M/41	AML without maturation/AML M1	46, XY[20]	p.Arg882His (R882H)	p.Trp288Cysfs*12 (W288C) (mutation A with TCTG insertion)	Negative	Negative
F/42	AML with maturation/AML M2	46, XX[20]	p.Arg885Trp (R885W), p.Val649Met	Negative	Negative	*IDH1*: p.Arg132Cys (R132C)
F/63	AML with maturation/AML M2	46, XX[20]	p.Arg882Cys (R882C), p. Glu545* (E545*)	Negative	Negative	Negative
M/58	Acute monocytic leukaemia/AML M5b	46, XY[20]	p.Arg882His (R882H)	p.Trp288Cysfs*12 (W288C) (mutation A with TCTG insertion)	Positive	Negative
F/54	AML with maturation/AML M2	46, XX[20]	p.Gly722Asp (G722D)	Negative	Negative	*IDH2*: p.Arg140Gln (R140Q)
F/57	AML without maturation/AML M1	46, XX[20]	p.Val636Leu (V636L)	p.Trp288Cysfs*12 (W288C) (mutation A with TCTG insertion)	Not done	Negative
F/50	Acute myelomonocytic leukaemia/AML M4	46, XX[20]	p.Trp795* (W795*)	p.Trp288Cysfs*12 (W288C) (mutation A with TCTG insertion)	Positive	Negative

**TABLE 2:** Co‐occurrence of gene mutations in acute myeloid leukaemia (AML) patients

		NPM1					FLT3‐ITD		
		WT	Mutant	*p*			WT	Mutant	*p*
*DNMT3A*	WT	41	13	.009	*DNMT3A*	WT	48	6	.002
	Mutant	16	17			Mutant	18	12	

		*IDH1/2*					*IDH1*		
		WT	Mutant	*p*			WT	Mutant	*p*
*DNMT3A*	WT	43	11	.293	*DNMT3A*	WT	50	4	.460
	Mutant	23	10			Mutant	29	4	

		*IDH2*					*TET2*		
		WT	Mutant	*p*			WT	Mutant	*p*
*DNMT3A*	WT	47	7	.508	*DNMT3A*	WT	47	7	.908
	Mutant	27	6			Mutant	29	4	

		*NPM1*					*NPM1*		
		WT	Mutant	*p*			WT	Mutant	*p*
*FLT3‐ITD*	WT	50	19	.008	*IDH1/2*	WT	45	21	.354
	Mutant	7	11			Mutant	12	9	

		*NPM1*					*NPM1*		
		WT	Mutant	*p*			WT	Mutant	*p*
*IDH1*	WT	53	26	.333	*IDH2*	WT	49	25	.743
	Mutant	4	4			Mutant	8	5	

		*IDH1*							
		WT	Mutant	*p*					
*IDH2*	WT	66	8	.213					
	Mutant	13	0						

Abbreviation: WT, wild type.

The *NPM1* mutations detected by NGS (30/87 or 34.5% patients) were successfully validated by conventional molecular methods. The majority of *NPM1* mutations (26/30, 86.7%) were type A mutations (insertion of TCTG).^27^ There were two patients (2/30, 6.7%) with type D (insertion of CCTG), one (1/30, 3.3%) was type B (insertion of CATG) and another (1/30, 3.3%) with the type DD‐3 (insertion of CAGA) mutations. Of these 30 patients *NPM1‐*mutated cases, 29 (29/30, 96.7%) had a normal karyotype. *NPM1* mutations were frequently concurrent with *FLT3‐ITD* mutations (*p* = .008) (Table 2).There were *FLT3‐ITD* mutations found in 18 of the 30 patients (18/87, 20.7% of all), of which 14 patients had a normal karyotype.

*IDH1* mutations were detected in 8 patients (8/87, 9.2%) and all cases had a normal karyotype‐AML (NK‐AML). All *IDH1* mutations were located in R132 with R132H mutations being the most common (5/8, 62.5%) and followed by R132C mutations (3/8, 37.5%). *IDH2* mutations were detected in 13 patients (13/87, 14.9%), 11 (84.6%) of whom had a normal karyotype. In 10 patients (10/13, 76.9%) *IDH2* mutations were located in R140 with all of being R140Q mutations. *IDH1* and *IDH2* mutations did not co‐exist in our cohort (Table 2).

## 4 DISCUSSION

In this study, we used a targeted NGS panel to sequence the whole exons of 120 genes known to be mutated in myeloid neoplasms, in 87 newly diagnosed Chinese adult patients with AML. Mutations were detected in 78/87 (89.7%) patients with an average of 1.9 mutations per patient. Of note, was that 60/87 (69%) patients with variants had NK‐AML. The most frequently mutated genes were similar to those previously reported Caucasian AML cohorts, including *DNMT3A*, *NPM1*, *IDH1/2* and *FLT3‐ITD*.^6^, ^11^, ^12^ We showed higher gene mutation frequencies than reported for other Chinese cohorts.^16^, ^17^, ^18^ This was seen for both *DNMT3A* (37.9% vs. 12.5%–14.0%) and *NPM1* (34.5% vs. 15.0%–15.9%) mutations.

We found *DNMT3A* was the most common mutated gene, present in 37.9% of patients, almost double the frequencies in other reports.^6^, ^11^, ^12^ Other Chinese studies^16^, ^17^, ^18^ had shown *DNMT3A* mutation frequencies of 12.5%–14.0% which were slightly less than the Caucasian populations and other Asian population countries (Korea: 17.5%; Japan: 19.0%; Singapore: 21.0%).^13^, ^14^, ^15^ Although the frequency of *DNMT3A* mutations was high, a single nucleotide change in R882 was commonly seen (39.4%), from arginine to histidine (R882H mutation, 69.2%) or cysteine (R882C, 23.1%), as reported in other studies.^12^, ^13^, ^28^ *DNMT3A* mutations seemed to co‐occur with *NPM1* (*p* = .009) and *FLT3‐ITD* mutations (*p* = .002). Among the AML patients with *DNMT3A* mutations, 6.1% of patients had concomitant *NPM1*, *FLT3‐ITD* and *IDH* mutations while 24.2% AML patients had triple mutations of *DNMT3A*, *NPM1* and *FLT3‐ITD*. These findings were in line with published reports on *DNMT3A* in patients with AML.^6^, ^11^, ^13^, ^29^, ^30^ The high frequencies of *DNMT3A* and *NPM1* mutations in our study may be due to the high incidence of normal karyotype (69.0%). However, those with *DNMT3A* mutations were not in the older age group, as the mean age of this cohort was 61 years. Further those with concurrent DNMT3A and NPM1 mutations (*n* = 17) also were not of older age (mean = 60.2 years). These findings therefore do not support those of previous reports^6^, ^13^, ^30^ that show that patients with *DNMT3A* mutations were generally older in age. A larger cohort is needed to further investigate these relationships and explore the different gene mutation frequencies are related to genetic background, lifestyle, environment and other factors.

*DNMT3A* is an epigenetic regulator catalyses DNA methylation in CpG islands and regulates the gene silencing processes.^31^ It is vital in normal haematopoietic stem cell differentiation^32^ and self‐renewal and its mutation produces a sufficient amount of preleukaemic stem cells which finally convert into AML.^33^ Mutations in *DNMT3A* and *IDH1/2*, genes that encode epigenetic modifiers, are present in the early pre‐leukaemic cells and these “founder” mutations can be implicated as functional components of AML evolution. These genes are frequently mutated in elderly patients with clonal haematopoiesis.^11^ *NPM1* mutations are regarded as secondary events and usually occur after *DNMT3A* and *IDH1* mutations. These suggest that development of AML will follow specific and ordered evolutionary processes. Recent studies showed that *DNMT3A* R882 mutation exerts a dominant‐negative effect. The mutant protein then interferes with the remaining normal *DNMT3A* to form active tetramers that reduces the enzyme activity and hypomethylation at specific cytosine‐guanine dinucleotides in early AML cells.^34^

NPM1 is a nuclear protein involving ribosome biogenesis, DNA repair and prevent apoptosis.^23^ The frequency of *NPM1* mutations was higher than in other Chinese cohorts (15.0%–15.9%).^16^, ^17^, ^18^ We found *NPM1* mutations in 34.5% of our patients with the majority (86.7%) being type A mutations (insertion of TCTG). Of these, 96.7% had a normal karyotype‐AML.

The combined frequency of *IDH1/2* mutations was 24.1%, in line with other publications.^6^, ^11^ *IDH1* mutations were found in 9.2% of the AML patients and all cases had a normal karyotype (NK‐AML). It has been reported that patients with *IDH1* mutations are commonly older. This was not the case in the present study where the mean age of patients with *IDH1/2* mutations was 60 years (range 42–85 years), consistent with the full cohort with mean age of 62 years. All *IDH1* mutations detected were single nucleotide change in R132 with R132H mutations being the most common (62.5%). Dang et al.^35^ showed that R132 *IDH1* mutations cause the encoded enzyme to acquire the novel ability to convert alpha‐ketoglutarate (α‐KG) to 2‐hydroxy‐glutarate (2HG). The increased cellular 2HG levels will cause inhibition of α‐KG‐dependent enzymes that are important for the demethylation of DNA.^36^, ^37^, ^38^ *IDH2* mutations were more common, detected in 14.9% of the AML patients with 84.6% having normal karyotype. The most common *IDH2* mutation caused changes of R140 with all cases were R140Q mutation. No patients had both *IDH1* and *IDH2* mutations, in keeping with previous reports^9^, ^14^, ^39^, ^40^ that these mutations are mutually exclusive. Studies^38^, ^41^, ^42^ showed that *IDH1* and *IDH2* seem to act as an epigenetic role in histone and DNA methylation. *IDH1* and *IDH2* mutations then cause a hypermethylation phenotype in leukaemia and inhibit haematopoietic stem cell differentiation.

Several studies^12^, ^13^, ^14^, ^16^, ^29^, ^30^ have shown that AML patients with *DNMT3A* mutations had poorer clinical outcomes compared with the wild‐type *DNMT3A*. *DNMT3A* mutations were also associated with worse survival for AML patients with a normal cytogenetic and those with an intermediate‐risk profile. For the unique subgroup of AML patients with concomitant *DNMT3A*, *NPM1* and *FLT3‐ITD* mutations, they had the poorest prognosis.^13^, ^29^ Since *DNMT3A*, *NPM1* and *FLT3* mutations belong to the three separate classes of mutations, this suggests the possible interaction between different classes of gene mutation in AML pathogenesis.

For our study, we only performed large‐scale gene sequencing on the leukaemia samples. Although the use of matched normal specimens may be important in the identification of recurrent variants, it is not necessary when a good filtering system has been established.^43^ We used “population frequency” approach which is the percentage of the samples with mutation in the database of sample sequenced in order to filter out common benign variants.^44^ This is important in filtering out the common germline polymorphisms and homopolymer‐related artefacts with particular high frequency.

In conclusion, simultaneous screening of multiple gene mutations using a 120‐gene targeted NGS approach has identified high frequencies of genomic variants in adult de novo AML. In addition, we have shown that *DNMT3A* is the most common mutation in Chinese AML patients. The frequency of both *DNMT3A* and *NPM1* mutations are higher than in other published studies of Chinese patients, three and twofold, respectively. *DNMT3A* mutations tended to co‐occur with *NPM1* and *FLT3‐ITD* mutations in patients with NK‐AML.

## CONFLICT OF INTEREST

The authors have stated explicitly that there are no conflicts of interest in connection with this article.

## AUTHOR CONTRIBUTIONS

**B.M.W.L.**: Conceptualization; methodology; investigation; formal analysis; resources; data curation; writing‐original draft; writing‐review and editing. **Y.L.K**.: Conceptualization; methodology; formal analysis; resources; writing‐original draft. **W.N.E.**: Conceptualization; methodology; validation; formal analysis; resources; supervision; project administration; funding acquisition; writing‐original draft; writing‐review and editing. **B.B.G.**: formal analysis. **J.A.J.M.**: formal analysis, manuscript drafting.

## ETHICS STATEMENT

The study protocol was approved by the Research Ethics Committee, Hospital Authority, Hong Kong. The Ethics Committee had waived the requirement for informed consent because archival buffy coat samples were used and all data had been fully anonymized

## Supporting information


**Appendix** S1. Supporting Information

**SUPPLEMENTARY TABLE 1** Clinical and molecular features of 87 adult Chinese patients with de novo AML

## Data Availability

The data that support the findings of this study are available from the corresponding author upon reasonable request.

## References

[1] Swerdlow SH , Campo E , Harris HL , et al. WHO Classification of Tumours of Haematopoietic and Lymphoid Tissues. IARC Press; 2008.

[2] Vardiman JW , Thiele J , Arber DA , et al. The 2008 revision of the World Health Organization (WHO) classification of myeloid neoplasms and acute leukemia: rationale and important changes. Blood. 2009;114:937‐951. 19357394 10.1182/blood-2009-03-209262

[3] Arber DA , Orazi A , Hasserjian R , et al. The 2016 revision to the World Health Organization classification of myeloid neoplasms and acute leukemia. Blood. 2016;127(20):2391‐2405. 27069254 10.1182/blood-2016-03-643544

[4] Betz BL , Hess JL . Acute myeloid leukemia diagnosis in the 21st century. Arch Pathol Lab Med. 2010;134:1427‐1433. 20923295 10.5858/2010-0245-RA.1

[5] Welch JS , Ley TJ , Link DC , et al. The origin and evolution of mutations in acute myeloid leukemia. Cell. 2012;150(2):264‐278. 22817890 10.1016/j.cell.2012.06.023 PMC3407563

[6] Cancer Genome Atlas Research Network , Ley TJ , Miller C , et al. Genomic and epigenomic landscapes of adult de novo acute myeloid leukemia. N Engl J Med. 2013;368:2059‐2074. 23634996 10.1056/NEJMoa1301689 PMC3767041

[7] Gilliland DG , Jordan CT , Felix CA . The molecular basis of leukemia. Hematology. 2004;2004:80‐97. 10.1182/asheducation-2004.1.80 15561678

[8] Link DC . Molecular genetics of AML. Best Pract Res Clin Haematol. 2012;25(4):409‐414. 23200536 10.1016/j.beha.2012.10.002 PMC3513694

[9] Patel JP , Gonen M , Figueroa ME , et al. Prognostic relevance of integrated genetic profiling in acute myeloid leukemia. N Engl J Med. 2012;366:1079‐1089. 22417203 10.1056/NEJMoa1112304 PMC3545649

[10] Estey EH . Acute myeloid leukemia: 2013 update on risk‐stratification and management. Am J Hematol. 2013;88(4):318‐327. 23526416 10.1002/ajh.23404

[11] Papaemmanuil E , Gerstung M , Bullinger L , et al. Genomic classification and prognosis in acute leukemia. N Engl J Med. 2016;374:2209‐2221. 27276561 10.1056/NEJMoa1516192 PMC4979995

[12] Ley TJ , Li D , Walter MJ , et al. DNMT3A mutations in acute myeloid leukemia. N Engl J Med. 2010;363(25):2424‐2433. 21067377 10.1056/NEJMoa1005143 PMC3201818

[13] Tan M , Ng IKS , Chen Z , et al. Clinical implications of DNMT3A mutations in a southeast Asian cohort of acute myeloid leukaemia patient. J Clin Pathol. 2017;70:669‐676. 28100593 10.1136/jclinpath-2016-204195

[14] Shin SY , Lee ST , Kim HJ , et al. Mutation profiling of 19 candidate genes in acute myeloid leukemia suggests significance of DNMT3A mutations. Oncotarget. 2016;7(34):54825‐54837. 27359055 10.18632/oncotarget.10240 PMC5342384

[15] Wakita S , Yamaguchi H , Ueki T , et al. Complex molecular genetic abnormalities involving three or more genetic mutations are important prognostic factors for acute myeloid leukemia. Leukemia. 2016;30:545‐554. 26488113 10.1038/leu.2015.288

[16] Lin PH , Li HY , Fan SC , et al. A targeted next‐generation sequencing in the molecular risk stratification of adult myeloid leukemia: implications for clinical practice. Cancer Med. 2017;6(2):349‐360. 28070990 10.1002/cam4.969 PMC5313641

[17] Han X , Li W , He N , et al. Gene mutation patterns of Chinese acute myeloid leukemia patients by targeted next‐generation sequencing and bioinformatics analysis. Clin Chim Acta. 2018;479:25‐37. 29309772 10.1016/j.cca.2018.01.006

[18] Wei H , Wang Y , Zhou C , et al. Distinct genetic alteration profiles of acute myeloid leukemia between Caucasian and eastern Asian population. J Hematol Oncol. 2018;11(1):18. 29427994 10.1186/s13045-018-0566-8 PMC5807853

[19] Hossfeld DK . World Health Organization classification of tumors: pathology and genetics of tumors of haematopoietic and lymphoid tissues. Ann Oncol. 2002;13:490‐491.

[20] Shaffer LG , Tommerup M . An International System for Human Cytogenetic Nomenclature. S. Karger AG; 2005.

[21] Shaffer LG , McGowan‐Jodan J , Schmid M . An International System for Human Cytogenetic Nomenclature. S. Karger AG; 2013.

[22] Dohner H , Estey EH , Amadori S , et al. Diagnosis and management of acute myeloid leukemia in adults: recommendations from an international expert panel, on behalf of the European LeukemiaNet. Blood. 2010;115:453‐474. 19880497 10.1182/blood-2009-07-235358

[23] Komanduri KV , Levine RL . Diagnosis and therapy of acute myeloid leukemia in the era of molecular risk stratification. Annu Rev Med. 2016;67:59‐72. 26473413 10.1146/annurev-med-051914-021329 PMC5701748

[24] Suzuki R , Onizuka M , Kojima M , et al. Prognostic significance of FLT3 internal tandem duplication and NPM1 mutations in acute myeloid leukemia in an unselected patient population. Int J Hematol. 2007;86:422‐428. 18192111 10.1007/BF02984000

[25] Huang Q , Chen W , Gaal KK , Slovak ML , Stein A , Weiss LM . A rapid, one step assay for simultaneous detection of FLT3/ITD and NPM1 mutations in AML with normal cytogenetics. Br J Haematol. 2008;142:489‐491. 18477048 10.1111/j.1365-2141.2008.07205.x

[26] Guo BB , Allcock RJ , Mirzai B , et al. Megakaryocytes in myeloproliferative neoplasms have unique somatic mutations. Am J Pathol. 2017;187(7):1512‐1522. 28502479 10.1016/j.ajpath.2017.03.009 PMC5500825

[27] Lit BMW , Kwong YL , Wong KF . Immunohistochemical detection of cytoplasmic nucleophosmin in formalin‐fixed paraffin‐embedded marrow trephine biopsies in acute myeloid leukaemia. J Clin Pathol. 2016;69(5):409‐414. 26500335 10.1136/jclinpath-2015-203175

[28] Yan XJ , Xu J , Gu ZH , et al. Exome sequencing identifies somatic mutations of DNA methyltransferase gene DNMT3A in acute monocytic leukemia. Nat Genet. 2011;43(4):309‐315. 21399634 10.1038/ng.788

[29] Loghavi S , Zuo Z , Ravandi F , et al. Clinical features of de novo acute myeloid leukemia with concurrent DNMT3A, FLT3 and NPM1 mutations. J Hematol Oncol. 2014;7:1. 25281355 10.1186/s13045-014-0074-4 PMC4197326

[30] Thol F , Damm F , Ludeking A , et al. Incidence and prognostic influence of DNMT3A mutations in acute myeloid leukemia. J Clin Oncol. 2011;29:2889‐2896. 21670448 10.1200/JCO.2011.35.4894

[31] Bestor TH . The DNA methyltransferases of mammals. Hum Mol Genet. 2000;9(16):2395‐2402. 11005794 10.1093/hmg/9.16.2395

[32] Challen GA , Suen D , Jeong M , et al. DNMT3A is essential for haematopoitic stem cell differentiation. Nat Genet. 2011;44(1):23‐31. 22138693 10.1038/ng.1009 PMC3637952

[33] Shlush LI , Zandi S , Mitchell A , et al. Identification of pre‐leukaemic haematopoietistem cells in acute leukaemia. Nature. 2014;506(7488):328‐333. 24522528 10.1038/nature13038 PMC4991939

[34] Dushyant K , Anurag M , Manoj KP , Sukanta N , Kandarpa KS . DNMT3A (R882) mutation features and prognostic effect in acute myeloid leukemia in coexistent with NPM1 and FLT3 mutations. Hemat Oncol Stem Cell Ther. 2018;11:82‐89. 10.1016/j.hemonc.2017.09.004 29079128

[35] Dang L , White DW , Gross S , et al. Cancer‐associated IDH1 mutations produce 2‐hydroxyglutarate. Nature. 2009;462:739‐744. 19935646 10.1038/nature08617 PMC2818760

[36] Rakheja D , Konoplev S , Medeiros LJ , Chen W . IDH mutations in acute myeloid leukemia. Hum Pathol. 2012;43(10):1541‐1551. 22917530 10.1016/j.humpath.2012.05.003

[37] Im AP , Sehgal AR , Carroll MP , et al. DNMT3A and IDH mutations in acute myeloid leukemia and other myeloid malignancies: associations with prognosis and potential treatment strategies. Leukemia. 2014;28(9):1774‐1783. 24699305 10.1038/leu.2014.124 PMC4234093

[38] Zhang X , Shi J , Zhang J , et al. Clinical and biological implications of IDH1/2 in acute myeloid leukemia with DNMT3Amut. Cancer Manag Res. 2018;10:2457‐2466. 30122995 10.2147/CMAR.S157632 PMC6084071

[39] Ribeiro AF , Pratcorona M , Erpelinck‐Verschueren C , et al. Mutant DNMT3A: a marker of poor prognosis in acute myeloid leukemia. Blood. 2012;119:5824‐5831. 22490330 10.1182/blood-2011-07-367961

[40] Meyer SC , Levine RL . Translational implications of somatic genomics in acute myeloid leukaemia. Lancet Oncol. 2014;15:e382‐e394. 25079101 10.1016/S1470-2045(14)70008-7

[41] Figueroa ME , Abdel‐Wahabo LC , et al. Leukemic IDH1 and IDH2 mutations result in a hypermethylation phenotype, disrupt TET2 function and impair hematopoietic differentiation. Cancer Cell. 2010;18(6):553‐567. 21130701 10.1016/j.ccr.2010.11.015 PMC4105845

[42] Wang M , Yang C , Zhang L , Schaar DG . Molecular mutations and their co‐occurrence in cytogenetically normal acute myeloid leukemia. Stem Cells Int. 2017;6962379:1‐11. 10.1155/2017/6962379 PMC5288537 28197208

[43] Carol B , Neff TL , Heinrich MC , et al. Combining highly multiplexed PCR with semiconductor‐based sequencing for rapid cancer genotyping. J Mol Diagn. 2013;15:171‐176. 23274167 10.1016/j.jmoldx.2012.09.003

[44] Singh RR , Patel KP , Routbort MJ , et al. Clinical validation of a next‐generation sequencing screen for mutational hotspots in 46 cancer‐related genes. J Mol Diagn. 2013;15:607‐622. 23810757 10.1016/j.jmoldx.2013.05.003

