# Supplementary material for: Mutation profile of acute myeloid leukaemia in a Chinese cohort by targeted next‐generation sequencing
Source: Cancer Rep (Hoboken). 2021 Oct 6;5(10):e1573. doi: 10.1002/cnr2.1573 (PMC9575498; doi:10.1002/cnr2.1573)
Supplement: Supplementary file 1 — Appendix S1. Supporting Information [file CNR2-5-e1573-s002.doc]

**Supplementary Appendix 1: Ion AmpliSeq Custom Panel includes the following 120 genes associated with aberrations in myeloid neoplasms:**

ABL1, ACSM2A, ASXL1, ATRX, BCOR, BCORL1, BIRC3, BOD1L1, BRAF, CACNA1E, CALR, CARD11, CBFBB, CBL, CBLB, CBLC, CDKN2A, CEBPA, CREBBP, CSF1R, CSF2, CSF3R, CTCF, CTNNA1, CUX1, CYP2D6, DAXX, DDX41, DNM2, DNMT1, DNMT3A, DNMT3B, EBF1, ECT2L, EED, EGFR, EP300, EPOR, ETV6, EZH2, FBXW7, FGFR1, FLT3, GATA1, GATA2, GATA3, GNAS, HRAS, IDH1, IDH2, IKZF1, IL-7R, ITGA8, JAK1, JAK2, JAK3, JARID2, KANSL1, KAT6A, KMD6A, KIT, KMT2A, KRAS, LUC7L2, MEF2B, MET, MLL2, MPL, MYB, MYC, MYCBP2, MYD88, NBEAL1, NF1, NF2, NFE2, NOTCH1, NPM1, NRAS, NTRK3, PAX5, PDGFRA, PDGFRB, PHF6, PIK3CA, PNTP11, PRPF40B, PRPF8, PTEN, PTPN11, RAD21, RAD50, RAF1, RB1, RELN, RUNX1, SETBP1, SETD2, SF1, SF3A1, SF3B1, SH2B3, SMC1A, SMC3, SOS1, SRSF2, SUZ12, TCF3, TCF3, STAG2, TCF3, TET2, TLR4, TP53, U2AF1, U2AF2, UGT1A1, WT1, XRCC2, ZRSR2.
