# Supplementary material for: Mutation profile of acute myeloid leukaemia in a Chinese cohort by targeted next‐generation sequencing
Source: Cancer Rep (Hoboken). 2021 Oct 6;5(10):e1573. doi: 10.1002/cnr2.1573 (PMC9575498; doi:10.1002/cnr2.1573)
Supplement: Supplementary file 2 — SUPPLEMENTARY TABLE 1 Clinical and molecular features of 87 adult Chinese patients with de novo AML [file CNR2-5-e1573-s001.doc]

**SUPPLEMENTARY TABLE 1**

**Clinical and molecular features of 87 adult Chinese patients with *de novo* AML**

| **Patient Gender/**  **Age (years)** | **WHO / FAB Diagnosis** | **Karyotype** | ***DNMT3A* Mutations** | ***NPM1* Mutations** | ***FLT3-ITD* Mutations** | ***IDH1/2* Mutations** |
| --- | --- | --- | --- | --- | --- | --- |
| F/54 | AML without maturation / AML M1 | 46, XX[18] | p.Arg882Cys  (R882C) | Negative | Positive (57 base pairs duplication) | *IDH1*:  p.Arg132His (R132H) |
| F/79 | AML with maturation / AML M2 | 46,XX,der(1)inv(1)(p36.1q21),t(1;3)  (q11;q21)[1]/46,sl, del(17)  (q12q23)[10]/46,sdl1, add(16)  (p11.1)[9]/46,XX[1] | Negative | Negative | Negative | Negative |
| F/74 | AML with myelodysplasia-related changes (complex karyotype) | 56,XX,+1,+3,+4, +6x2,+idic(7)  (p11.1),+8x2,+9, -13,?t(13;17)  (q11;q25),+15,idic(18)(p11.2),+19,  +21 [9]/55,idem,idic(7)(p11.1), +del(7)(p12),-13, der(17) ?t(13;17)(q11;q25)[2]/46,XX[5] | Negative | Negative | Negative | Negative |
| M/50 | AML with maturation/ AML M2 | 45,X,-Y[10]/46,XY, del(9)(q22)[2]/  46,XY[6] | Negative | Negative | Negative | Negative |
| M/85 | AML with maturation / AML M2 | 47,XY,+8[24] | Negative | Negative | Negative | Negative |
| M/69 | AML with maturation / AML M2 | 45,X,-Y,t(8;21) (q22;q22)[10] | Negative | Negative | Negative | Negative |
| M/53 | AML with maturation / AML M2 | 46,XY,t(2;11)(q31; p15)[20] | Negative | Negative | Positive  (45 base pairs duplication) | Negative |
| F/91 | AML with myelodysplasia-related changes (MDS-related cytogenetic abnormality) karyotype) | 44,XX,+X,del(5)(q13q31), dic(17;20)(p11.1;q11.2),-18,-19,  add(19)(p13.3)[3]/44,XX,del(5)  (q13q31),dic(17;20)(p11.1;q11.2),  -19,add(19)(13.3),?dic(21;?)  (p13;?)[14]/45,XX,del(5)  (q13q31),+11,der(14;16)(q10;p10), dic(17;20)(p11.1;q11.2)[2] | Negative | Negative | Negative | Negative |
| M/74 | AML with maturation / AML M2 | 47,XY,+21[17]/46,XY[2] | Negative | Negative | Negative | Negative |
| M/48 | Acute myelomonocytic leukaemia /AML M4 | 46, XY[22] | p.Pro904leu (P904L) | Negative | Negative | Negative |
| M/78 | AML with maturation / AML M2 | 47,XY,+8[16] | Negative | Negative | Negative | *IDH2*:  p.Arg140Gln (R140Q) |
| F/61 | AML without maturation / AML M1 | 46,XX[11] | Negative | p.Trp288Cysfs*12 (W288C)  (Mutation A with TCTG Insertion) | Positive  (84 base pairs duplication) | *IDH2*:  p.Arg140Gln (R140Q) |
| M/78 | AML with maturation / AML M2 | 46,XY[19] | p.Arg882His (R882H) | Negative | Positive  (8 base pairs duplication) | Negative |
| M/65 | AML with maturation / AML M2 | 42~45,XY,-8,-10,-18,-20[cp9]/  46,XY[9] | Negative | Negative | Negative | Negative |
| F/51 | AML with myelodysplasia-related changes (multilineage dysplasia) | 46,XX[15] | Negative | p.Trp288Cysfs*12 (W288C)  (Mutation A with TCTG Insertion) | Negative | *IDH1*: p.Arg132His (R132H) |
| F/44 | Acute monoblastic leukaemia/AML M5a | 46,XX,+1,der(1;21)(q10;q10)[1] /47,idem,+8[15]/47,idem,+mar[4]/46,XX[1] | Negative | Negative | Negative | Negative |
| F/54 | AML with maturation / AML M2 | 47, XX,+?15[2]/46, XX[18] | p.Arg882His (R882H) | Negative | Negative | *IDH2*:  p.Arg140Gln (R140Q) |
| F/46 | AML without maturation / AML M1 | 46, XX[20] | p.Ser714cys (S714C) | Negative | Negative | *IDH2*: p.Arg172Lys (R172K) |
| F/41 | AML without maturation / AML M1 | 46, XX[20] | Negative | p.Trp288Cysfs*12 (W288C)  (Mutation DD-3 with CAGA Insertion) | Negative | Negative |
| F/70 | Acute myelomonocytic leukaemia /AML M4 | 46, XX[19] | p.Lys299Asnfs (K299N) | Negative | Negative | Negative |
| F/31 | AML without maturation / AML M1 | 45,XX,-7[3]/45,idem,t(4;13)  (p10;p10)[8]/46,XX[4] | Negative | Negative | Negative | Negative |
| F/20 | AML with maturation / AML M2 | 45,X,-X,t(2;21;8) (q37;q22;q22), del(9)(q22)[19]/46,XX [1] | Negative | Negative | Negative | Negative |
| F/53 | AML without maturation / AML M1 | 47, XX, t(8; 21) (q22; q22),+15[19] | p.Lys299Asnfs (K299N) | Negative | Positive  (39 base pairs duplication) | Negative |
| F/50 | AML with maturation / AML M2 | 50,XX,+X,+del(1)(q42),del(5)  (q13q33),+11,+1,+19x2, -20,-22[15] /50,XX,+X,+del(1)(q42) del(5)  (q13q33), +16,+19,+20, dic(20;22)  (q11.1;q13),-2,+mar[2]/51~52,XX, +X,+del(1)(q42), del(5)(q13q33),  +11,+16,+19, +20x2,dic(20;22)  (q11.1;q13)x2 [cp2]/46,XX[1] | Negative | Negative | Negative | Negative |
| M/68 | AML without maturation / AML M1 | 46, XY[20] | Negative | Negative | Negative | Negative |
| F/72 | Acute monocytic leukaemia /AML M5b | 46, XX[20] | p.Arg882 Pro (R882P) | p.Trp288Cysfs*12 (W288C) (Mutation A with TCTG Insertion) | Positive  (18 base pairs duplication) | Negative |
| M/61 | AML with minimal differentiation / AML M0 | 47,XY,+13[18]/46,XY[1] | Negative | Negative | Negative | Negative |
| F/43 | Acute myelomonocytic leukaemia / AML M4 | 46, XX[20] | p.Arg882 His (R882H) | p.Trp288Cysfs*12 (W288C)  (Mutation A with TCTG Insertion) | Positive  (48 base pairs duplication) | Negative |
| M/52 | AML without maturation / AML M1 | 46, XY, t(2, 12)(p.23;q24.1)[20] | p.Cys710Tyr (C710Y) | p.Trp288Cysfs*12 (W288C)  (Mutation A with TCTG Insertion) | Positive  (42 base pairs duplication) | Negative |
| M/77 | AML without maturation / AML M1 | 46,XY[7] | Negative | p.Trp288Cysfs*12 (W288C)  (Mutation A with TCTG Insertion) | Negative | Negative |
| F/49 | AML with myelodysplasia-related changes (multilineage dysplasia) | 46,XX[21] | Negative | p.Trp288Cysfs*12 (W288C)  (Mutation D with CCTG Insertion) | Negative | Negative |
| M/45 | AML without maturation / AML M1 | 47,XY,del(9)(q12q34),+i(21)(q10) [17]/46,XY[3] | Negative | Negative | Negative | Negative |
| M/73 | AML with myelodysplasia-related changes (multilineage dysplasia) | 47,XY,+8[13]/46,XY[8] | Negative | Negative | Negative | Negative |
| M/77 | AML with minimal differentiation / AML M0 | 45,XY,der(12;17)(q10;q10)[1]/  46,XY[19] | Negative | Negative | Negative | Negative |
| M/66 | AML without maturation/ AML M1 | 46, XY[18] | p.Glu545* | p.Trp288Cysfs*12 (W288C)  (Mutation A with TCTG Insertion) | Positive  (87 base pairs duplication) | *IDH2*: p.Arg140Gln (R140Q) |
| M/55 | AML with maturation / AML M2 | 46,XY,der(6)t(3;6)(q12;p22)[7]/ 47,XY,+8[2]/46,XY[11] | Negative | Negative | Negative | Negative |
| M/80 | AML with myelodysplasia-related changes (multilineage dysplasia) | 46, XY [20] | Negative | Negative | Positive  (26 base pairs duplication) | Negative |
| M/81 | AML with maturation / AML M2 | 47,XY,+mar[2]/47,XY,+21[1]/  46,XY[17] | Negative | Negative | Negative | Negative |
| F/42 | AML with maturation / AML M2 | 46,XX[18] | Negative | Negative | Negative | *IDH2*: p.Arg172Lys(R172K) |
| M/80 | AML without maturation / AML M1 | 46, XY [20] | Negative | Negative | Negative | *IDH2*: p.Arg140Gln (R140Q) |
| M/45 | Acute myelomonocytic leukaemia /AML M4 | 46, XY [20] | p.Arg882Cys (R882C) | p.Trp288Cysfs*12 (W288C)  (Mutation A with TCTG Insertion) | Negative | *IDH2*: p.Arg140Gln (R140Q) |
| F/62 | AML without maturation / AML M1 | 46, XX [21] | p.Arg882His (R882H) | Negative | Negative | *IDH2*: p.Arg172Lys  (R172K) |
| M/59 | AML with maturation / AML M2 | 46, XY,t(10;11) (q23; p15)[1]/46,  idem,add(6)(p25)[19] | p.Arg882His (R882H), p.Lys299Asnfs (K299N) | Negative | Positive  (27 base pairs duplication) | Negative |
| F/60 | AML with maturation / AML M2 | 46, XX[20] | Negative | p.Trp288Cysfs*12 (W288C)  (Mutation B with CATG Insertion) | Negative | *IDH1*: p.Arg132His (R132H) |
| M/85 | AML with maturation / AML M2 | 46,XY[22] | Negative | Negative | Negative | *IDH2:* p.Arg140Gln (R140Q) |
| F/33 | AML with minimal differentiation / AML M0 | 47,XX,+4[2]/46,XX[20] | Negative | Negative | Negative | Negative |
| F/77 | AML with myelodysplasia-related changes (multilineage dysplasia) | 46, XX[20] | Negative | Negative | Negative | Negative |
| F/65 | Acute monocytic leukaemia / AML M5b | 46, XX[20] | Negative | p.Trp288Cysfs*12 (W288C)  (Mutation D with CCTG Insertion) | Negative | Negative |
| F/46 | AML with maturation/ AML M2 | 46, XX[19] | Negative | Negative | Negative | Negative |
| F/70 | AML with maturation/ AML M2 | 46, XX[20] | p.Ser714Cys (S714C), p.Glu545* | Negative | Negative | Negative |
| F/55 | AML with maturation/ AML M2 | 46, XX[20] | Negative | Negative | Negative | Negative |
| M/85 | AML with maturation/ AML M2 | 47,XY,+i(13)(q10)[7]/46,XY[18] | Negative | Negative | Negative | Negative |
| F/80 | AML without maturation/ AML M1 | 45,XX,del(1)(p32),-6,+15,-16,-17,  -18,-20,+mar1,+mar2, +mar3[24] | Negative | Negative | Negative | Negative |
| F/78 | AML without maturation/ AML M1 | 46, XX[20] | p.Arg882His (R882H), P.Glu545* | p.Trp288Cysfs*12 (W288C)  (Mutation A with TCTG Insertion) | Positive | *IDH1*: p.Arg132His (R132H) |
| M/65 | Acute myelomonocytic leukaemia/AML M4 | 46, XY [20] | P.Glu545* | p.Trp288Cysfs*12 (W288C)  (Mutation A with TCTG Insertion) | Negative | *IDH1*: p.Arg132His (R132H) |
| M/78 | AML without maturation / AML M1 | 46, XY [20] | Negative | Negative | Negative | Negative |
| M/72 | AML with maturation / AML M2 | 46, XY [20] | p.Trp893Val, p.Glu545* | Negative | Negative | Negative |
| F/36 | Acute myelomonocytic leukaemia / AML M4 | 46, XX [20] | Negative | p.Trp288Cysfs*12 (W288C)  (Mutation A with TCTG Insertion) | Positive | Negative |
| F/87 | AML with myelodysplasia-related changes (multilineage dysplasia) | 46, XX [18] | p.Glu545* | Negative | Negative | Negative |
| M/50 | AML without maturation / AML M1 | 46, XY [20] | p.Arg882His (R882H), p.Glu545*,  p.Trp893Valfs | p.Trp288Cysfs*12 (W288C)  (Mutation A with TCTG Insertion) | Negative | Negative |
| F/44 | AML with maturation / AML M2 | 46, XX [20] | Negative | Negative | Negative | Negative |
| M/67 | AML with maturation / AML M2 | 46, XY [20] | Negative | Negative | Negative | *IDH1*: p.Arg132Cys (R132C) |
| M/80 | AML without maturation / AML M1 | 46, XY [19] | p.Cys911Tyr  (C911Y),  p.Glu545* | p.Trp288Cysfs*12 (W288C)  (Mutation A with TCTG Insertion) | Negative | Negative |
| F/49 | AML without maturation / AML M1 | 46, XX [20] | p.Glu545* | p.Trp288Cysfs*12 (W288C)  (Mutation A with TCTG Insertion) | Not done | Negative |
| F/82 | AML with myelodysplasia-related changes (multilineage dysplasia) | 46, XX [20] | p.Glu545* | p.Trp288Cysfs*12 (W288C)  (Mutation A with TCTG Insertion) | Negative | Negative |
| F/65 | AML with maturation / AML M2 | 46, XX [20] | Negative | Negative | Negative | Negative |
| M/81 | AML with maturation / AML M2 | 46, XY [20] | p.Glu545* | Negative | Negative | Negative |
| F/40 | AML with maturation/ AML M2 | 46, XX [20] | Negative | Negative | Negative | Negative |
| M/88 | Acute myelomonocytic leukaemia / AML M4 | 46, XY [20] | p.Ser638Cys (S638C) | p.Trp288Cysfs*12 (W288C)  (Mutation A with TCTG Insertion) | Positive | Negative |
| M/76 | AML without maturation/ AML M1 | 46, XY [20] | Negative | Negative | Positive | Negative |
| M/63 | Acute myelomonocytic leukaemia / AML M4 | 46, XY [20] | Negative | p.Trp288Cysfs*12 (W288C)  (Mutation A with TCTG Insertion) | Negative | Negative |
| M/50 | AML without maturation / AML M1 | 46, XY [20] | p.Thr503Asnfs (T503N) | p.Trp288Cysfs*12 (W288C)  (Mutation A with TCTG Insertion) | Negative | Negative |
| M/42 | AML with maturation / AML M2 | 46, XY [20] | Negative | p.Trp288Cysfs*12 (W288C)  (Mutation A with TCTG Insertion) | Positive | *IDH2:* p.Arg140Gln (R140Q) |
| M/82 | AML without maturation / AML M1 | 46, XY [19] | Negative | Negative | Negative | Negative |
| M/30 | AML with maturation / AML M2 | 46, XY [20] | Negative | Negative | Negative | Negative |
| M/50 | AML with maturation / AML M2 | 46, XY [20] | Negative | p.Trp288Cysfs*12 (W288C)  (Mutation A with TCTG Insertion) | Negative | Negative |
| M/60 | AML with myelodysplasia-related changes (multilineage dysplasia) | 46, XY [20] | Negative | p.Trp288Cysfs*12 (W288C)  (Mutation A with TCTG Insertion) | Negative | *IDH2:* p.Arg140Gln (R140Q) |
| F/62 | AML without maturation / AML M1 | 46, XX [20] | Negative | Negative | Negative | Negative |
| M/41 | AML without maturation / AML M1 | 46, XY [20] | p.Arg882His (R882H) | p.Trp288Cysfs*12 (W288C)  (Mutation A with TCTG Insertion) | Negative | Negative |
| F/42 | AML with maturation / AML M2 | 46, XX [20] | p.Arg885Trp  (R885W),  p.Val649Met | Negative | Negative | *IDH1*: p.Arg132Cys (R132C) |
| M/83 | AML with maturation / AML M2 | 46, XY [18] | Negative | Negative | Negative | *IDH1*: p.Arg132Cys (R132C) |
| F/63 | AML with maturation / AML M2 | 46, XX [20] | p.Arg882Cys  (R882C),  p.Glu545*  (E545*) | Negative | Negative | Negative |
| M/58 | Acute monocytic leukaemia / AML M5b | 46, XY [20] | p.Arg882His (R882H) | p.Trp288Cysfs*12 (W288C)  (Mutation A with TCTG Insertion) | Positive | Negative |
| F/54 | AML with maturation / AML M2 | 46, XX [20] | p.Gly722Asp  (G722D) | Negative | Negative | *IDH2:* p.Arg140Gln (R140Q) |
| F/57 | AML without maturation / AML M1 | 46, XX [20] | p.Val636Leu  (V636L) | p.Trp288Cysfs*12 (W288C)  (Mutation A with TCTG Insertion) | Not done | Negative |
| M/87 | Acute myelomonocytic leukaemia / AML M4 | 46, XY [19] | Negative | p.Trp288Cysfs*12 (W288C)  (Mutation A with TCTG Insertion) | Negative | Negative |
| F/50 | Acute myelomonocytic leukaemia / AML M4 | 46, XX [20] | p.Trp795* (W795*) | p.Trp288Cysfs*12 (W288C)  (Mutation A with TCTG Insertion) | Positive | Negative |
